# Influence of a sexism and sexual harassment prevention course on medical students’ perceptions of and responses to problematic situations

**DOI:** 10.1186/s12909-023-04902-0

**Published:** 2023-12-08

**Authors:** Yves Evéquoz, Lauriane Pichonnaz, Julie Dubois

**Affiliations:** 1 Institute of Family Medicine, Faculty of Science and Medicine, University of Fribourg, Fribourg, Switzerland; 2 Gender and Health Unit, Department of ambulatory care, University Center for Primary Care and Public Health (Unisanté), University of Lausanne, Lausanne, Switzerland

**Keywords:** Sexism, Sexual harassment, Medical students, Medical education, Prevention course

## Abstract

**Background:**

Issues of sexism and sexual harassment within the medical field are widespread. The aim of his study was to describe the influence of a training course on the prevention of sexism and sexual harassment on medical students’ perceptions and responses to problematic situations in the workplace.

**Methods:**

We performed a secondary analysis of the transcripts of 16 medical students’ interviews. The data were initially gathered as part of an external evaluation of the course. We decided to further explore these with a focus on potential changes about sexism and sexual harassment awareness induced by the course. Data were analyzed using qualitative thematic analysis.

**Results:**

Most medical students were aware of the existence of sexism and sexual harassment in the medical environment before the course but were not always able to delineate what could be considered as such or not. The course allowed them to broaden their ability to identify problematic situations, that were somewhat “trivialized” before, and to set a lower cut-off when judging what constitutes sexism or sexual harassment. It also provided them with tools on how to react when confronted to these situations, as well as resources to seek help. However, students stressed the importance of such courses also being offered to medical staff higher up in the hierarchy, as they are the ones in a position of power.

**Conclusions:**

The course helped students to better identify and react to sexism and sexual harassment. However, given students’ lack of power within the hierarchy, efforts to prevent sexism and sexual harassment must be undertaken at an institutional level to allow for change in the system as a whole.

## Background

Issues of sexism and sexual harassment (S/SH) in the workplace are serious and widespread [1], including in the medical field [2, 3]. While both men and women may experience situations of S/SH, these situations are more likely to happen to women [3–8]. For example, Jagsi et al. [4] showed that 66% of female clinical researchers reported experiencing gender discrimination (vs. 10% of men), and 30% sexual harassment (vs. 4% of men). The perpetrators are not only “insiders” [6, 7] but also patients and patients’ families [8, 9]: in some cases, they even represent 49% of all incidents reported [9]. Experiences of S/SH are associated with negative effects on mental and physical health, as well as on working conditions and private life [1, 2, 6, 10].

S/SH can occur at all levels of the medical field. As stated in the report by the National Academies of Sciences, Engineering, and Medicine : “mistreatment is commonplace in all levels of the medical hierarchy, especially among medical school students, interns, and residents in all specialties” [3]. If we focus on the specific situation of female medical students, they are notably more likely than their colleagues in other faculties to experience some form of sexual harassment [3]. Reasons that could explain it are manifold, such as male dominated structures, students’ dependency on superiors and staff, hierarchical institutions, and potentially isolating training environments [3, 11, 12] In addition, these situations are often underreported, due to concerns about personal consequences and lack of effective institutional response [11, 13, 14].

In an attempt to address this issue, a course on the prevention of S/SH was held at two Swiss medical schools (Lausanne and Fribourg universities), the aim of which was to “ (1) identify situations of S/SH as targets, authors, or bystanders and (2) act on problematic situations by using tools, institutional or legal policies, and procedures for reporting SH, as taught during training” [15]. The course consisted of two parts, with some minor differences between the two universities. The first part relied on the tools from the Theater of the Oppressed [16], a kind of theatre that focuses on situations of oppression, giving participants the opportunity to express themselves and to work with the group to find ways of improving the situation. The second part gave theoretical inputs about S/SH at the workplace (legal, social, individual) and its impact on health, and presented institutional resources available to the students. The course consisted of a single teaching session lasting between 2.5 and 3.5 h depending on the University where it took place [15].

The target audience for this course was third-year bachelor and first-year master students, depending on the medical school. The teaching took place before the immersion in the clinic, i.e. before hospital internship rotations began. Indeed, students in clinical years are at greater risk of experiencing S/SH compared to pre-clinical students [13].

This course was set up in the context of a growing awareness about the issue of S/SH in these two medical schools, which also led to the implementation of poster campaigns and a student association, CLASH, whose aim is to fight against S/SH and collect testimonies from medical students.

As we will see later, the preventive measures of the course and the actions of CLASH need to be complemented and expanded to enable systemic change. In this article, our analysis and objectives primarily focus on comparing students’ knowledge about and reaction to S/SH before and after the course.

## Methods

The course was subject to an external evaluation aimed at assessing its relevance, its implementation, as well as its effect on students. The implementation process of the course, its content, and results of the evaluation are described elsewhere [15, 17]. In this article, we performed a secondary analysis of the students’ interviews conducted by the team responsible for the external evaluation of the course. Initially, the students were selected based on a random sampling ensuring a balanced representation of teaching sessions between Lausanne and Fribourg. As around two-thirds of course participants were women, particular care was taken to reproduce the gender distribution among interviewees. The evaluation team invited the students to participate by e-mail. The interviews were then conducted by phone with the students consenting to participate. At that time, 17 semi-structured interviews were conducted. The interviews lasted around 45 min each [17]. The interviews were only one part of the evaluation, which also included interviews with stakeholders, questionnaires, as well as observations of the courses. Given the scope of the evaluation, which was to measure the effects of the course and the achievement of its objectives, students’ interviews were not extensively analyzed. We thus decided to further explore these with a focus on potential changes about S/SH awareness induced by the course.

The Cantonal Commission for Ethics in Human Research of Vaud (CER-VD) (Req-2022-00917) waived the need for ethics approval for this study as no health-related data were collected. However, all procedures performed in this study were in accordance with the Swiss Federal Act on Research involving Human Beings [18] and with the 1964 Helsinki Declaration and its later amendments or comparable ethical standards [19].

After informing the students about the purpose of the present analysis and seeking their written informed consent to reuse their interviews, the evaluation team provided us with the anonymized transcripts. Only students’ gender and university name were available to the authors of the present article, to preserve students’ confidentiality. 16 out of 17 students consented to the reuse of their interview transcripts, 7 from the University of Fribourg and 9 from the University of Lausanne. Six of them identified as male, and 10 as female.

A thematic analysis of the transcripts was conducted using a qualitative data analysis software: MAXQDA 20.4.0 (VERBI Software). The first transcript was coded in parallel by two members of the team (YE and JD) and then compared to reach an agreement over the codes used and to cooperatively develop a codebook. The codebook was then applied to all the other transcripts by YE. The codebook was adapted iteratively as new codes emerged from the data. New codes and changes in the definitions of codes were discussed with JD for agreement. JD cross-checked all the transcripts to ensure validity and rigor as well as consensus in the application of the codebook. Similar codes were then merged into subthemes and themes derived from the interview guide.

## Results

An overview of the thematic structure of our results is provided in Table 1. We identified four main themes: awareness of S/SH before the course, experiences of S/SH, knowledge gained from the course, and call for further improvements.

**Table 1:** Overview of the thematic structure of results

Themes	Sub-themes	Codes
Awareness of S/SH before the course	Level of knowledge	Low knowledge Well informed
	Type of reactions	No reaction
		Avoidance Trusted resources
Experiences of S/SH	Confrontation to S/SH	A widespread issue No confrontation Personal experiences
	Specificities of the healthcare environment	Specificities of the healthcare environment
	Challenges to reaction	Not knowing how to react Fear of consequences
	Trivialization	Trivialization
Knowledge gained from the course	Importance of the course	An important topic Awareness-raising course Preventive role of the course
	Messages retained	Available resources Importance of reacting to S/SH
	Identification of S/SH	Setting a lower cut-off Profile of perpetrators
Call for further improvements	Recurrence of the course over time Teaching other healthcare professionals Institutional measures	Recurrence of the course over time Teaching other healthcare professionals Institutional measures

S/SH: sexism and sexual harassment

### Awareness of sexism/sexual harassment before the course

This first theme regroups what students knew or believed about S/SH before the course.

#### Level of knowledge

Although most students had heard of the students’ association (CLASH) or seen the campaigns against S/SH at their University, about one third of them admitted to not knowing a lot about the subject before the course: “*Apart from the fact that it exists, I would say [my knowledge was] weak. I had no idea about the forms of harassment, about the contexts of harassment that might be more common or more favorable, about the frequency of harassment, I had no idea, also about the solutions to harassment as well as all the legal aspects concerning harassment (…). I didn’t know anything. No one I knew had told me about what had happened to them and nothing had happened to me so I had no personal experience, direct or indirect, that I could think of*.” (Female student 5).

In the continuity of the latter statement, most had heard of it but were unclear about its definition: “*In the sense that yes, we know what harassment is, but we don’t really know what it means to harass. In the sense that for me, before, when I talked about harassment it was only with physical contact. However, as I learned during the course, it’s also related to psychology, it’s not only something physical*.” (Male student 4).

Another student thought that sexism did not occur at such level of education: “*I knew that it existed in the street but as I told you before: the higher the level of education, the less sexism there is. But the numbers you gave us speak for themselves, and therefore we have the proof that it is not true*” (Male student 1).

Two thirds of the students, in contrast, were already well informed and aware of the issue before the course, especially female students: “*I mean, I can’t say I’m an expert at all, but it’s something I’m interested in, so I’ve already informed myself a little bit anyway. So, I think I already knew some things. […] My roommate […] gave me references and books that I looked at a little bit, the Internet too of course but I don’t have any specific pages, and a lot of discussions too, with my roommate or other colleagues*” (Female student 6).

#### Types of reaction to S/SH

When asked how they would have reacted to S/SH situations before the course, students mentioned that it was not clear to them. Most evoked avoidance strategies: “*I think that I had - until now, honestly, I would have had more avoidance techniques, rather than confronting too much. Maybe now I would dare to confront the problem a bit more I would say and maybe talk to my superior to tell him that it’s not going well. But before, I was more likely to avoid the situation that would make me feel uncomfortable*” (Female student 4). One student underlined the difference between daring to react as a witness or as a target: “*I would have a much easier time reacting as a witness than as a target. I think I’ve always reacted when I think things are not right, whether it’s sexism or something else, I usually react. But for myself it’s always more complicated because I think “maybe he’s right actually, I am just a woman”. But if I witness it, if it’s not against me, I have more courage to react*.” (Female student 7).

Others evoked some potential resources and said they would have contacted someone they could trust, like family, colleagues or the students’ association CLASH. However, most were not aware of available institutional or legal resources: “*I would have gone to someone I trusted, among my friends or doctors with whom I get on well, and we would have thematized the thing, but that’s about it*.” (Male student 2).

### Experiences of S/SH

#### Confrontation to S/SH

As the interview guide focused primarily on the course and its importance in the context of the medical curriculum, students mostly talked about experiences lived within the hospital. However, the interviews revealed that S/SH can appear everywhere and at any time: “*I do a lot of music, I work in festivals, at the level of the teacher-student relationship, there can be things like that, so I don’t know if there are more in the hospital environment*” (Female student 9). Similarly, when asked about the importance of having such a course a student responded: “*Very important. First of all, because in the health care environment, there are problems of harassment, but also because there are problems in everyday life, so it is good to be able to do prevention in general*” (Male student 3).

Only six students stated that they had never been confronted to situations of S/SH, two of them were men: “ *Well, as a white male who speaks French in a hospital where they speak French, I don’t think I was really the target of sexual harassment. At least I didn’t experience it as such*” (Male student 2). Other interviewees gave several examples of situations where they experienced S/SH, either as witnesses or targets. The first quotation refers to a situation concerning another female assistant doctor, after a meeting held by webcam: “*The assistant doctor who had to manage this little meeting had her mobile phone and it filmed the part from the bottom of her neck to her navel, but it didn’t film the rest. And during the coffee break afterwards, the [male] teacher came and said to her: “It’s not that I don’t like to see this part of your anatomy, but next time you could perhaps put your face on it”. It’s little remarks like that, but when you know a bit about the character, it makes everyone laugh a little. But I think it made her a bit uncomfortable (…). I honestly couldn’t see myself saying something to the teacher in the middle of the cafeteria in front of everyone. Everybody kind of snickered, nobody said anything*.” (Female student 4).

Another student evoked a situation where she was the target: “*It was with the head doctor, again in the operating room. (…) I attended, it was a hip operation, and there it was already- he doubted very much that I could do this and that’s always the subject, “am I strong enough for this?” And they always told me, “now pull like a man” or “here, it’s like at home, the man says when it’s over”, all that. But because I had a good relation with the head doctor I dared to tell him, I don’t remember what I said exactly, but it was in a kind of ironic way, “wow great, there is real emancipation or feminism in ortho[pedics], it’s true what they told me!“ They laughed and it wasn’t super clear that I was mad but I was able to say I did not agree with it*” (Female student 3). Students also gave example where patients where the perpetrators: “*They told me that they didn’t want to talk to a woman, that they wanted to talk to a man […]. They didn’t think that I was a doctor, it was immediately “can you get me a glass of water? can you get me this or that?“ […]. Or they would say to me “ah I like the little trainees” well that kind of thing; suddenly they would talk to me about their erections, well anyway… There are a thousand little things like that, which accumulate, but it happens to everyone all the time*” (Female student 6).

#### Specificities of the healthcare environment

Students considered that there are favorable conditions in the health care environment that explain the occurrence of S/SH: the closed environment and proximity, the fact that it is a highly hierarchical environment, as well as long-lasting clichés about women in medicine: “*Well, promiscuity is sometimes imposed, or often imposed. Then there’s also the hierarchy which is very present, well still present, and also the whole image of the nurse, precisely we always talk about nurses in the female form. It’s the image of the sexy nurse in a white coat, well that’s it*” (Female student 2).

Regarding the special atmosphere of the hospital, it has been described as “barracks like” by some students: “*But it’s true that when you work in the hospital, you see that in the higher positions there is a majority of men, and you hear quite a few stories… And you also feel it in certain specialties, that there is a bit of a side, not macho, but a bit of a barracks attitude. I can live with it, but I think that as a woman it’s a bit difficult, so I think it’s a good thing that there is that [the course]*” (Female student 4).

#### Challenges to reaction

The conditions described above did not make it easy to react for the students who were confronted to S/SH: “*When we arrive in the system, we are the newcomers and the ones who are the most vulnerable, the most likely to be affected by it, also the ones who have the least weapons to defend ourselves against it*” (Male student 5). Indeed, the interviews showed that most students do not react or do not know how to react to S/SH: “*On the other hand, my approach to knowing how to react was null. That is to say, I thought the workshops [from the course] were great, because I wouldn’t have known how to react if I were a victim of harassment, or if I wanted to help one of my fellow students, I wouldn’t have known how to react at all*” (Male student 6). Furthermore, non-reaction was explained by perceived difficulties in denouncing S/SH, such as fear of retaliation and students’ low hierarchical position: “*If I myself am an assistant and he is the head of the department where I have always wanted to practice, where I want to be hired afterwards, then it gives an additional complexity. If I’m willing to say to myself, “Okay, I’m going to be sanctioned for that because I’ve opened my mouth”-in the end it’s so intramural in this hospital environment that if I say something, it’s the chief’s word against mine. And so, if I say something that he doesn’t like, I’m bound to be afraid of losing my job*” (Male student 1).

#### Trivialization

Non-reaction might also be explained by a trivialization of behaviors that can be considered as sexism or sexual harassment, either by witnesses or by the targets themselves. As one male student stated: “*We often don’t realize it, we think: “Oh, it’s nothing”, but it’s true that, just calls in the street, or insistent looks, I think everyone has seen that at least once*” (Male student 3). Another particularly interesting example was found when one student minimized her previous experiences with the expression “nothing more”: “*Personally, I don’t have a history of sexual harassment any more than someone in the street who says “Good evening, Miss” or something like that. Or a hand that’s a little bit out of place, it’s happened, but nothing more*” (Female student 9).

Trivialization was also observed in students minimizing the magnitude of the problematic: “*And what annoys me is when people make the connection between “Yes, there are chief doctors, they are like that” and “All men are like that”, it’s something that is extremely hurtful for the majority of men, because it seems to me, and I hope - maybe you’ll contradict me - but I hope that it’s the noisy minority that really does that (…). It’s just a very small minority that does things like that*” (Male student 1).

### Knowledge gained from the course

This category was unsurprisingly the most important one in our analysis, as this was the focus of the evaluation.

#### Importance of the course

All students agreed on the importance of this course. A majority of the students underlined the awareness-raising role of the course: “*Often when you’re a man, (…) you’re a little less aware of this issue, but you just have to talk to girls, they’ve all often experienced situations. But we don’t see it too much, so it’s clear that we have to be interested to see that it’s really present and that it exists. So it’s good that there’s a course that makes us aware of it a little bit to take a first step in this direction*.” (Male student 3). Some students even recognized suffering from some bias, like in the following example: “*Well, to say to myself “I had the prejudice that it was in South America that there was the most sexism, whereas there may be just as much here, but I didn’t realize it”. Facing up to one’s prejudices, that’s what it’s all about*.” Female student 1)

Some students also underlined the preventive role of the course and the importance of insuring safer work environments for the future: “*We are going to be the next generation, and sooner or later, we will be asked to set an example, and especially to be reference persons for the victims of sexual harassment. And it is important that we have talked about it early, and that we can react in an adequate and correct way to break the cycle and really set a good example for it*.” (Male student 2). As one student stated, preventing S/SH is also important in terms of well-being: “*In itself, simply by logic, if we want to take care of the people around us, we have to be well too. And if we are in an environment where we are disturbed by psychological violence or other things, well that doesn’t make any sense (…). And a healthcare environment should be a healthcare environment for everyone, including the caretakers*” (Female student 1). Finally, such a course may help prevent becoming a perpetrator, for example once in a superior hierarchical position: “*I would say to be careful not to act like that ourselves. Not to create harassment ourselves without realizing it. Or being sexist without realizing it. It’s pretty easy to make sexist remarks that can hurt someone when you didn’t mean to. So, I think it’s important not to forget that we can also be the aggressor, without any bad thoughts, but we have to be careful about that*.” (Female student 7).

#### Messages retained

The first key message retained was that students are not alone and that there are resources available to them, like students’ association and institutional resources: “*Don’t forget that you are not alone in this and that you can ask for help in many different places. Whether it’s our friends, CLASH, human resources, there are many ways not to be alone*” (Female student 7). However, if the institutional intention of combating S/SH was clear, for example the zero-tolerance claimed by the two medical schools, it was not the case for the way it would be put into practice: “*The Faculty has- they said [the teachers] - zero tolerance. But what that means exactly, they didn’t really know. (…). Anyway, I took it as a principle, I’m satisfied with it, it’s already very good.*” (Female student 5).

The second key message retained was the importance of reacting, either as a witness or a target. All students agreed with the fact that it is important to react when witnessing S/SH, either by intervening directly or at least by acknowledging it by talking to the target afterwards: “*If you see something, you have to do something, no matter what it is and no matter if it is during or after. Even before. Before, to prevent it from happening. During, to reduce the time it happens. After, if you couldn’t do anything before and during, to take care of the victim afterwards, to give her/him an outlet so that she/he can validate her/his pain. I think that in all three stages of action, something can and should be done*” (Male student 1). As potential targets, students underlined the importance of facing the perpetrator or to at least talk about the situation with someone else: “*Even talking about it with someone else. Yeah anyone around us, either a supervisor, but maybe a woman, or a man, depending on our gender, well refer to someone of our gender who can maybe understand, refer to us and maybe has been through it, who will know what to do.*” (Female student 7). Nevertheless, the interviews showed that it remained difficult to react, even after following the course, mostly because of the fear for one’s own career.

#### Identification of S/SH

Most students were able to identify patent sexism or sexual harassment before the course. However, more “subtle” forms were not always identified as such. Indeed, “subtlety” of S/SH (referring to the act) implies a form of subjectivity on the side of the potential target (referring to the perception): “*For me it’s a limit that is quite personal because we are not all sensitive to the same things and the same words will not trigger the same reactions or the same gestures in everyone. For me it’s more a question of feeling. And that’s what I found very good in the course; you just have to say things, say how you feel and say that you feel it’s inappropriate, without it being a box to tick like “it was at such and such a lumbar level so that means it was inappropriate”, no. It was us who felt that way*” (Female student 9). Similarly, some students identified as being problematic situations that were somewhat internalized as being normal before: “*I realized that there are situations that are a little subtler. So, that I have already experienced things that you could say “this is sexism”, or “this is harassment”. [Do you have any examples that you remember?] Well, typically whistling, which I perceived as part of our society, as part of being a girl. It’s not very nice but it’s okay. Or being publicly hit on in a place where I don’t feel like it or when I don’t feel like it*” (Female student 8). In general, students learned to put a lower cut-off when defining a behavior as being appropriate or not: “*Now I would say I would put the cut-off much lower. Before I would have said “Yes, well, okay. That’s inappropriate, but maybe it’s not yet sexual harassment”. Whereas now, it may not be much in the end, but in my opinion it’s enough to tip over into harassment. So, it’s my cut-off point that has moved*” (Male student 6). Finally, two students mentioned having learned about the profile of harassers and how it changed their representations : “*The play, especially, highlighted the fact, which is perhaps not the case in all forms of harassment, that it can come from close friends, colleagues, etc., who are not necessarily hierarchically superior. That it may also happen in totally incongruous situations, by people that we would not imagine [doing so]. (…). I’m telling you this because I didn’t have much knowledge or experience, so for me it was really a wake-up call, all the way through*.” (Female student 5).

### Call for further improvements

#### Recurrence of the course over time

Although interviews showed that students overall found this course to be very useful, some stressed that it was not enough, and that there should be more practice or that the course should be repeated and given at different moments throughout the curriculum: “*We should approach the subject more regularly, because I really liked the course we had, but on the other hand I think that in one afternoon, we won’t be able to integrate everything (…). After a few months, I would say that since I haven’t been confronted with this situation, I am as lost as before again. And I think that a regularity like that is very, very important*” (Female student 6).

#### Teaching other healthcare professionals

In addition to it, the students mentioned the possibility that not only the students should follow the course, but also the whole medical staff in order to change the system in its globality: “*It would be an idea to do this same course for physicians, senior physicians, chief physicians, medical staff, who could either be victims or perpetrators or witnesses. This awareness [towards S/SH] is new to this whole generation, all these people who work in the hospital, I don’t know how much awareness they have (…).*” (Female student 5).

#### Institutional measures

Finally, students pointed out the need for clear institutional measures that guarantee that problematic situations can be denounced without fear for one’s own career: “*I really think that the main thing would be (…) that there be a position from the university or the hospital that says that this is unacceptable and that there will be consequences when it happens. Not that it be “Oh yes, well, we avoid it” (…). And then really that we can assure the victims that there will be no repercussions, that they won’t have to go somewhere else to continue their studies or whatever*” (Male student 3).

## Discussion

Most medical students were aware of the existence of S/SH in the medical environment before the course, but a third were not clear about how to define it or delineate what can be considered sexism or sexual harassment. When confronted to it, they would have confided to trusted persons (colleagues or friends) or avoided problematic situations. These avoidance strategies are common in the medical learning environment, as several studies have pointed out [11, 20, 21].

Regardless of their ability to define sexism or sexual harassment, the majority of students had experienced or witnessed problematic situations, which is unsurprising given the prevalence of this issue in the medical context [2, 3, 11, 20, 22, 23]. The interviews revealed that, before the course, female students were in general more likely to identify S/SH. Indeed, “privilege is often invisible to those who have it, whereas bias and discrimination are readily apparent to those who experience it” [24]. If women are better at identifying these biases, they also often suffer more from the interiorization of implicit gender-career bias [25]. Most of the examples given by the students fall into the category of what has been called “everyday sexism” [26]. Everyday sexism is made up of repeated small references, jokes or inappropriate micro-attitudes, which can make it almost imperceptible. Everyday sexism is thus mostly constituted of microaggressions that can be subdivided into 4 groups: microinsults (e.g.: a female doctor mistaken for a nurse), micro assaults (e.g.: men are better physicians than women), microinvalidations (e.g: negating someone’s experience by calling them oversensitive) and environmental microaggressions (e.g: auditoriums named only after male physicians) [27]. The perpetrators of this form of sexism often do not realize that their behavior is discriminatory [27, 28]. Everyday sexism thus results in maintaining pervasive beliefs such as that women are less career oriented, more sensitive or less competent than men [29–31]. In addition, the subtlety of everyday sexism might explain that some students mentioned to have never been confronted to situations of sexism or sexual harassment, as they were maybe unable to identify these situations as such.

Students pointed to particular conditions conducive to the occurrence of S/SH in the hospital context, such as male-dominated, closed, and highly hierarchical environments. These particular conditions, in addition to dependency relationships between students and their hierarchy, and lack of institutional responses have already been highlighted as facilitating S/SH [1, 3, 12, 14, 20, 22]. These conditions made it difficult for students to react when confronted to problematic situations, mostly because their low hierarchical position made them feel vulnerable to retaliation. This fear of personal repercussions, along a certain trivialization of these situations, has been underlined in other studies as being key reasons for not denouncing S/SH [7, 11, 13, 20], thus leading to underreporting of S/SH [7, 13, 20, 32, 33]. Interviews in our study revealed that students were now more aware of available resources in the institution and that reacting to situations of sexism or sexual harassment was considered important. The course gave them tools to react, especially as witnesses, which is very important as witnesses may provide meaningful support to targets and are in a more comfortable position to react [24, 27].

In addition, interviews showed that in general, the course allowed to broaden students’ ability to identify S/SH, in particular everyday sexism, notably in situations that were somewhat “trivialized” before the course (“lower cut-off”). Everyday sexism is more difficult to identify because it does not clearly match the prototypical conceptions of what constitutes S/SH and is thus not readily identified as prejudice [34]. Broadening one’s ability to identify S/SH helps to embrace the definition of sexism as an ideological system “that dichotomizes and ranks genders by postulating the superiority of the category of men over the category of women” [35]. This is of high importance given that “subtle”, yet frequent forms of sexism or sexual harassment appear to be as harmful as overt yet punctual forms of sexism or sexual harassment (like sexual coercion) [1]. Failing to identify everyday sexism perpetuates gender biases that are pervasive to the medical environment (and society in general), while on the contrary, the ability to recognize them may help students identify the hidden curriculum perpetuating these biases and participate to institutional changes towards a more equal and equitable environment [25, 36]. Hafferty et al. [37] defined the hidden curriculum as “the attitudes and values conveyed, most often in an implicit and tacit fashion, sometimes unintentionally, via the educational structures, practices, and culture of an educational institution”. Moreover, at a personal level, this ability may help diminish the interiorization of implicit, and often unconscious, gender biases, which may have implications on several levels. First, it could enhance access to traditionally gendered specialties, such as surgery or obstetrics, to all students whatever their gender. Indeed, it has been shown that “gendered experiences in clinical practice influence the career choices of individual doctors and reduce gender diversity in the medical workforce” [31]. Second, it may help students identify their own biases, instead of internalizing them as normal, which can help them avoid perpetuating these and become perpetrators themselves. Similarly, identifying situations as sexist can encourage confrontation, even if the students’ position in the institutional hierarchy and fear of retaliation makes this a challenging process. Bystanders in particular, as already mentioned, have in important role to play in denouncing these situations. Indeed, the lack of reaction in the face of sexist situations by bystanders reinforces the idea that S/SH is socially acceptable [3, 34].

However, although students underlined the importance of the course to prevent S/SH, they deemed it not enough to change the system in its globality, given their lack of power within the hierarchy. It is thus critical that this type of preventive course is also made available (if not mandatory) to medical staff at all levels across the hierarchy, as has been advocated by many [1, 3, 13, 20, 24, 32, 38]. Cultivating safe environments with a zero-tolerance policy, as well as adequate prevention, at all hierarchy levels requires an inclusive transformation of the field of medicine, which is difficult, but absolutely possible and paramount [1, 10, 23, 39].

This is, to our knowledge, the first study that examines the influence of such an interactive course on students’ perceptions of S/SH. The results showed that the course helped students better identify and react to S/SH, whether as targets or bystanders, which constitutes a step towards a wider institutional shift regarding these issues and the constitution of safer learning and working environments. Nevertheless, this study has several limitations. First, the interview guide used for the evaluation of the course was not solely designed to capture changes in students’ perceptions of S/SH following the course but was also aimed at improving its structure and content. This may have prevented a more in-depth understanding of students’ perceptions regarding S/SH. However, this secondary analysis still revealed important changes in perceptions, as well as valuable insights into the importance of better identifying S/SH. This observation should prompt further research into the impact of such training on the responses to and identification of S/SH. Second, as the interviews were only one within several data collected to perform the evaluation, their number was relatively low. However, the analysis revealed that thematic saturation was reached. In addition, our data are consistent with those present in numerous other works about S/SH in the medical curriculum. As such courses are being put in place in other medical schools, it would be interesting to further evaluate their effects, to confirm the results presented here.

## Conclusions

The interviews showed the course had a good influence on understanding the mechanisms of S/SH, especially by broadening the students’ definitions that were before sometimes limited to the most visible aspects of S/SH. Courses on S/SH prevention should be offered in all medical schools in order to give students tools to address problematic situations. However, students alone cannot change the system. It is therefore essential that training institutions have a clear institutional policy of zero tolerance for sexism and sexual harassment, completed with regular mandatory courses on S/SH prevention, and that awareness of these issues is raised at all levels in order to witness a sustainable change.

## Data Availability

Raw qualitative datasets analyzed during the current study are not publicly available since consent for sharing data was not granted by participants; deidentified data may be available in French from the corresponding author on reasonable request.

## List of abbreviations

S/SH: sexism and sexual harassment
